# Risk Factors and Outcomes of *Klebsiella pneumoniae* Infection Before and After Allogeneic Hematopoietic Cell Transplantation

**DOI:** 10.3389/fmed.2020.608165

**Published:** 2021-02-04

**Authors:** Eleni Gavriilaki, Ioanna Sakellari, Thomas Chatzikonstantinou, Despina Mallouri, Ioannis Batsis, Eleni Katsifa, Stergios Papadimitriou, Alkistis Panteliadou, Eirini Baldoumi, Christos Demosthenous, Zoi Bousiou, Varnavas Constantinou, Damianos Sotiropoulos, Achilles Anagnostopoulos

**Affiliations:** ^1^Hematology Department - BMT Unit, G. Papanicolaou Hospital, Thessaloniki, Greece; ^2^Microbiology Department, G. Papanicolaou Hospital, Thessaloniki, Greece

**Keywords:** *Klebsiella pneumoniae*, allogeneic hematopoietic cell transplantation, graft-vs.-host disease, treatment-related mortality, overall survival

## Abstract

**Objectives:** *Klebsiella pneumoniae* carbapenemase (KPC)–producing *K. pneumoniae* (KPC-Kp) emerge as a major healthcare concern worldwide. Despite the significance of infections before and after allogeneic hematopoietic cell transplantation (alloHCT), the burden of KP infections has not been extensively evaluated.

**Methods:** We studied the incidence, risk factors, and outcomes of consecutive alloHCT recipients with Kp isolates before and after alloHCT.

**Results:** Among 424 patients who underwent alloHCT in 2008–2018, we studied two groups: those with Kp isolates before (group 1, 52 patients) and those with Kp isolates after alloHCT (group 2, 66 patients). prE-transplant infections were associated with post-transplant infections (*p* = 0.010), despite secondary prophylaxis. KPC-Kp was isolated in 29% of group 1, and 80% of group 2. Both groups were characterized by a significant burden of moderate–severe acute graft- vs.-host disease (GVHD) [cumulative incidence (CI) of 44.5 and 61.9%, respectively] and severe chronic (CI of 56.7 and 61.9%). Kp infections and GVHD were independent predictive factors of treatment-related mortality (TRM) in both groups.

**Conclusions:** Our study highlights the significant impact of Kp infections on TRM, with GVHD consisting an important underlying factor. As prophylactic measures did not improve rates of post-transplant infections, innovative interventions need to be further investigated to address this major healthcare concern.

## Introduction

Infections remain a major determinant of morbidity and mortality post–allogeneic hematopoietic cell transplantation (alloHCT) (1). AlloHCT recipients are at high risk of bacteremia early after transplantation because of two major insults to their innate immune system. First, these patients have rather prolonged neutropenia after receipt of their conditioning regimen and thus lack the first and most important phagocytes to combat bacterial infections (2). Second, their conditioning regimen leads to marked gastrointestinal (GI) mucositis, and thus, the integrity of their mucosal barrier is damaged. These two key insults establish a high-risk setting for bacteremia caused by enteric organisms and for severe complications from these infections. Therefore, bloodstream infections (BSIs) occur in 20 to 50% of alloHCT recipients, especially during neutropenia, and have been associated with poor morbidity and mortality (3–6).

Among Enterobacteriaceae, *Klebsiella pneumoniae* carbapenemase (KPC)–producing *K. pneumoniae* (KPC-Kp) are almost always carbapenem resistant (7). Carbapenemase-producing (KPC) Kp have emerged as a major healthcare concern worldwide (8). Despite the significance of BSIs before and after alloHCT, their burden has not been extensively evaluated. The problem has been recognized by a few studies in the transplantation field with mixed populations of autologous and allogeneic HCT recipients (7, 9–12). Interestingly, significant concerns have been raised regarding the feasibility of alloHCT in these patients (10, 11). Given the lack of studies in patients with Kp before and after alloHCT, we aimed to determine the incidence, risk factors, and outcomes of Kp infections in alloHCT recipients.

## Methods

### Study Population

We retrospectively studied consecutive adult patients who underwent alloHCT in our center in 2008–2018. Eligibility criteria included Kp isolates before or after alloHCT and patients' written informed consent to participate in the study. Patients with isolates only in rectal swabs were excluded.

We performed a retrospective review of data in our prospectively acquired database of HCT patients treated at our JACIE (Joint Accreditation Committee-International Society of Cell and Gene Therapy/ISCT & European Society for Blood and Marrow Transplantation/EBMT)–accredited unit meeting eligibility criteria. Patient data including details of the transplantation procedure, disease status, response rates, toxicity, survival time, and time to progression were extracted. Our institutional review board approved this study, and all patients gave a written informed consent in accordance with the Helsinki Declaration.

Patients were transplanted according to standard EBMT indications and standard operating procedures of our JACIE-accredited center, as previously described (5, 13–15). Assessment and grading of acute graft-vs.-host disease (GVHD) was performed according to criteria suggested by Glucksberg et al. whereas chronic GVHD was assessed and graded according to the criteria of Sullivan et al. (16, 17). Disease phase at transplant was categorized in three groups: first complete remission (early), other complete remission (intermediate), or advanced stage. Unrelated and haploidentical transplant recipients received low dose of rabbit ATG (thymoglobulin 5 mg/kg) as part of the conditioning, as previously described (18, 19).

### Infection Prevention and Control

Our facilities are equipped with HEPA-filtered isolation rooms to prevent in-hospital acquisition of airborne pathogens. Nurses, visitors, and all staff are carefully trained on infection control measures such as contact precautions and intensified hygienic measures in patients with pre-transplant isolation. It is our policy to test every patient with previous Kp colonization or infection with baseline cultures, rectal swabs, sputum stool, blood, and urine. Colonization is defined as the isolation of the microorganism from any non-sterile body site in the absence of clinical signs or symptoms of disease. Patients with colonization or infection pre-transplant received secondary prophylaxis during transplant.

All patients received supportive treatment against bacterial, fungal, and viral infections. Trimethoprim-sulfamethoxazole was used as prophylaxis for *Pneumocystis jirovecii* infection, and preemptive treatment for cytomegalovirus and Epstein–Barr virus reactivation was also administered according to close molecular monitoring. Ciprofloxacin prophylaxis was used as prophylaxis in neutropenic patients. Patients with previous Kp colonization or infection received secondary prophylaxis with antibiotics based on antibiotic *in vitro* sensitivity testing in aplastic period. In case of low-grade fever of unknown origin despite secondary prophylaxis, prompt empirical treatment was initiated. Antibiotic sensitivity testing for newer agents, such as ceftazidime-avibactam and ceftolozane-tazobactam, has been implemented since 2018 and has therefore not been included in the present analysis. Anti-infectious agents are administered in adequate dosing for immunocompromised patients. Leukocyte engraftment was defined as the first of three consecutive days with neutrophil count >0.5 × 10^9^/L, and platelet engraftment as the first of three consecutive days with counts >20 × 10^9^/L without transfusion.

### Statistical Analysis

Data were analyzed using the statistical program SPSS 22.0 (IBM SPSS Statistics for Windows, version 22.0. Armonk, NY: IBM Corp). To address our research question, we divided our population into two groups: those with Kp isolates before (group 1) and those with Kp isolates after alloHCT (group 2). As there is overlap between these two groups (patients who developed infections before and after alloHCT), no direct comparisons between groups were performed. Continuous variables were described as median and range and categorical variables as frequencies. Patient-, disease-, and transplant-related variables were compared using χ^2^ statistics for categorical variables and Mann–Whitney for continuous variables. Probabilities for relapse, treatment-related mortality (TRM), and GVHD were calculated using the cumulative incidence (CI) estimator to accommodate competing risks (CI of competing events and Gray test, and Fine–Gray proportional hazard regression for competing events) (20). Kaplan–Meier estimates were used to calculate the probability of overall survival (OS). Multivariate analysis was performed using Cox proportional hazards model for OS. Statistical analysis included the following factors: age, gender, disease phase at transplant (early, intermediate or advanced), donor (sibling, unrelated, or haploidentical), HLA mismatch, graft source [peripheral blood stem cells (PBSCs) or bone marrow], conditioning (myeloablative or reduced intensity), occurrence and time of pre-transplant and post-transplant Kp infections, KPC-Kp, moderate–severe acute (II–IV) and extensive chronic GVHD, relapse, TRM, and OS. Level of statistical significance was defined at 0.05.

## Results

### Study Population

Among 424 transplanted patients, we studied 52 patients with Kp isolates before (group 1) and 66 patients with Kp isolates after alloHCT (group 2). Table 1 presents basic patients' characteristics. None of the studied pre-transplant and transplant factors has been associated with Kp infections. Despite secondary prophylaxis that was administered in all patients with previous Kp infections, pre-transplant infections were associated with post-transplant Kp infections (*p* = 0.010). In other words, the percentage of pre-transplant infections was significantly higher in patients with post-transplant infections compared to those without (26 vs. 9%, *p* = 0.010). If the analysis is limited only to KPC-Kp infection or excludes KPC-Kp infection, this result is no longer significant.

**Table 1:** Patients' pre-transplant characteristics.

**Characteristics**	**Group 1 (*n* = 52)**	**Group 2 (*n* = 66)**
**Age, years (median, range)**	42 (17–42)	45 (17–64)
**Disease type (*****n*****)**		
AML	22	32
ALL	21	18
Lymphoma	4	6
MDS	2	4
Other	3	5
**Disease phase (*****n*****)**		
Early	29	43
Intermediate	13	12
Advanced	10	11
**Previous lines of treatment (median, range)**	4, 1–11	2, 1–11
**Conditioning (*****n*****)**		
Myeloablative	45	55
Reduced intensity	7	11
**Graft source (*****n*****)**		
Peripheral blood stem cells	47	58
Bone marrow	5	8
**Donor (*****n*****)**		
Sibling	19	23
Matched (8/8)/mismatched (7/8) unrelated	27/2	31/4
Haploidentical	3	8

*AML, acute myeloid leukemia; ALL, acute lymphoblastic leukemia; MDS, myelodysplastic syndrome*.

### Group 1

Among 52 patients with Kp detection before alloHCT, isolates were found in cultures of blood (42), stools (28), urine (18), and sputum (12), leading to Kp infections in all patients. KPC-Kp were reported in 12 patients (29% of Kp detections and 3% of total HCT recipients). Pre-transplant Kp infections occurred at a median of 145 (52–289) days before transplant.

**Figure 1:**
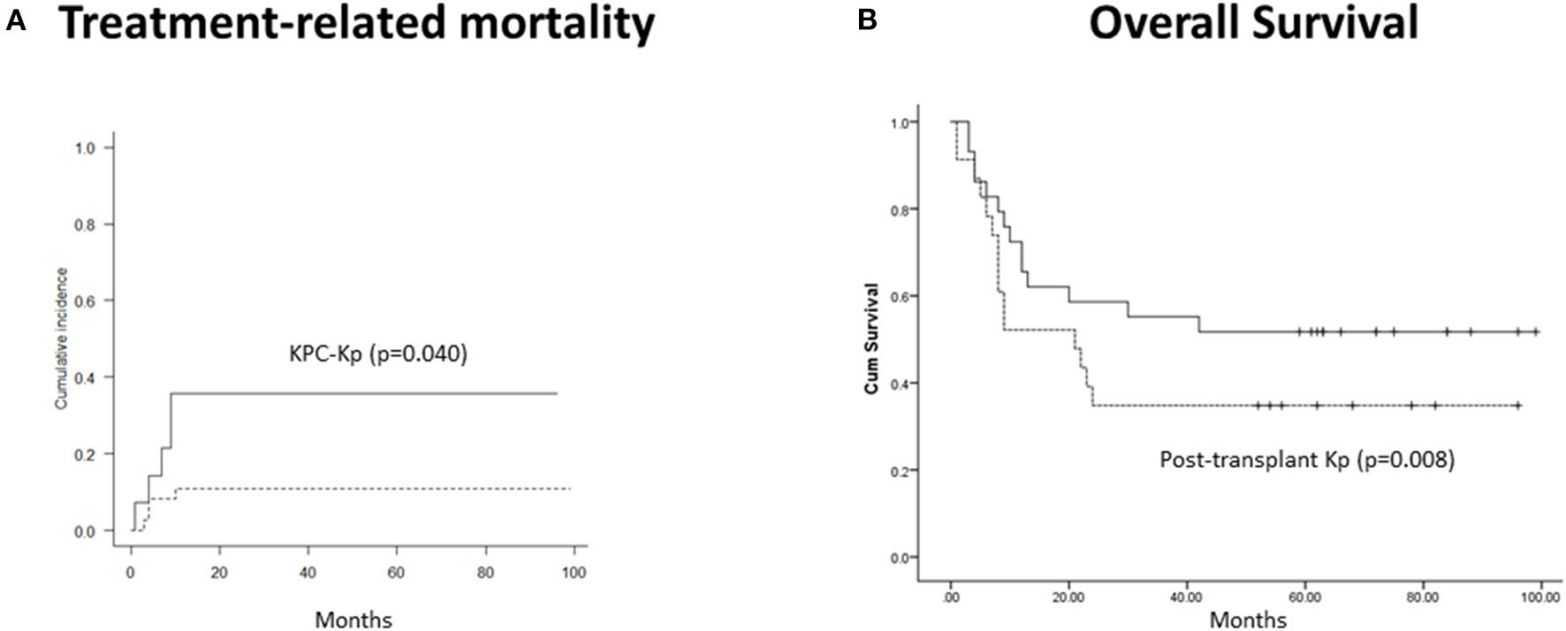
Outcomes in patients with Kp isolates before alloHCT (*n* = 52). **(A)** Treatment-related mortality was significantly higher in patients with KPC-Kp detection (*p* = 0.040). **(B)** Overall survival was significantly lower in patients with post-transplant Kp detection (*p* = 0.008). KP, *Klebsiella pneumoniae*; KPC, *Klebsiella pneumoniae* carbapenemase; alloHCT, allogeneic hematopoietic cell transplantation; Cum, cumulative.

With a median follow-up of 23.5 months (range, 1–99 months), CI of moderate–severe (grades II–IV) acute GVHD was 44.5%, and extensive chronic, 56.7%. Two-year CI of TRM was 14.3% and was independently predicted by the isolation of KPC-Kp (*p* = 0.040, Figure 1A) and chronic GVHD (*p* < 0.001). OS was associated with disease phase at transplant (*p* = 0.017), post-transplant infections (*p* = 0.034) and acute GVHD (*p* = 0.013). In the multivariate model, only post-transplant Kp infections independently predicted OS (β = 9.042, *p* = 0.008, Figure 1B).

### Group 2

Among 66 patients with Kp detection after alloHCT, isolates were found in cultures of blood (49), stools (32), urine (28), and sputum (17), leading to Kp infections. Pre-transplant Kp infections were evident in 17/66 patients (26%), and 12/17 were attributed to KPC-Kp. Pre-transplant infection was associated only with the development of moderate–severe acute GVHD (*p* = 0.016). Despite specific prophylaxis, KPC-Kp was also isolated in 52 patients post-transplant (80% of group 2 and 12% of alloHCT recipients), including all patients with pre-transplant infections. Post-transplant infections occurred at median 52 (5-376) post-transplant days.

**Figure 2:**
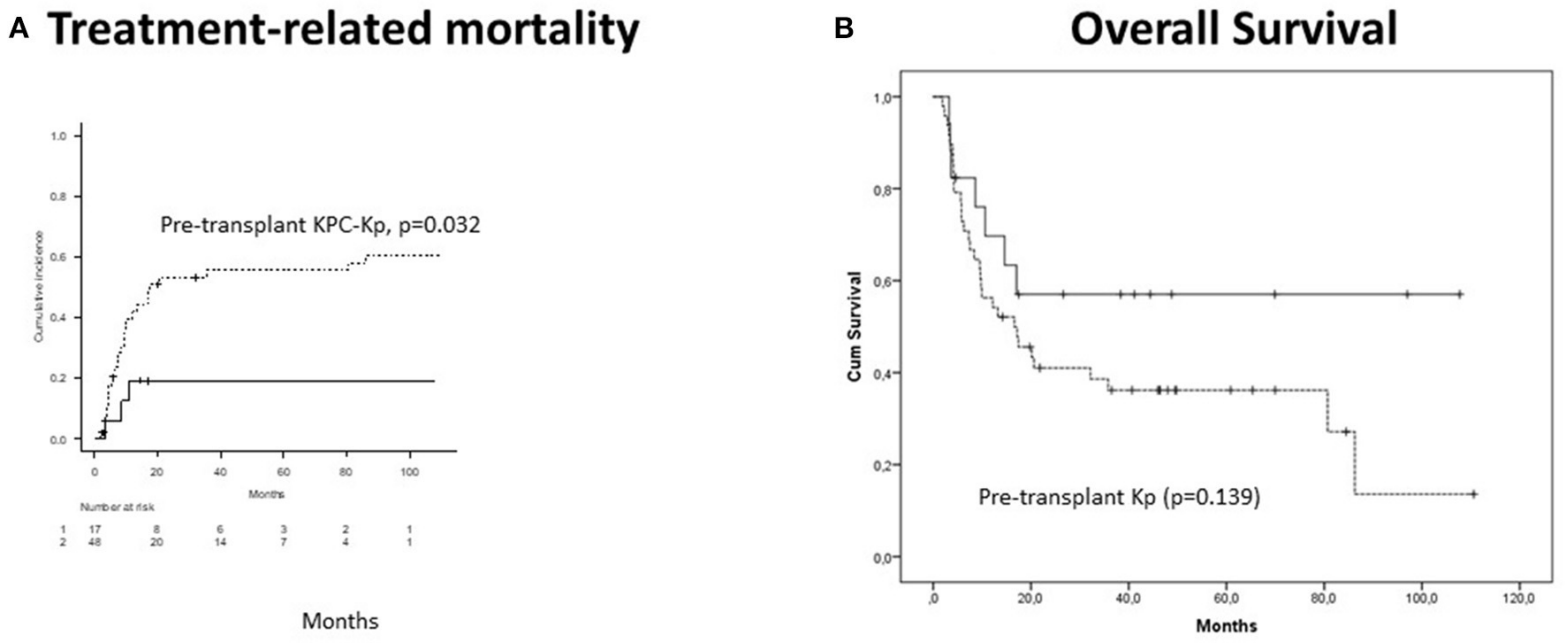
Outcomes in patients with Kp isolates after alloHCT (*n* = 66). **(A)** Treatment-related mortality was significantly higher in patients with pre-transplant KPC-Kp detection (*p* = 0.032). **(B)** Overall survival was not significantly lower in patients with pre-transplant Kp detection (*p* = 0.139). KP, *Klebsiella pneumoniae*; KPC, *Klebsiella pneumoniae* carbapenemase; alloHCT, allogeneic hematopoietic cell transplantation; Cum, cumulative.

CI of acute GVHD was 61.9% and was associated with pre-transplant irrespectively of KPC-Kp (*p* = 0.025) and KPC-Kp (*p* = 0.021) infections (pre-transplant or post-transplant). Chronic GVHD CI reached 58.5% and was associated with previous lines of treatment (*p* = 0.001) and phase at transplant (*p* = 0.005). Five-year CI of TRM was 47% and was independently predicted by the pre-transplant KPC-Kp infection (*p* = 0.032, Figure 2A) and acute GVHD (*p* = 0.018). With a median follow-up of 17 months (range, 2–110 months), OS reached 41.2% at 5 years. OS was not significantly lower in patients with pre-transplant Kp detection (*p* = 0.139, Figure 2B). In the multivariate analysis, OS was associated with disease phase at transplant (*p* = 0.009).

## Discussion

Our study highlights the significant impact of Kp infections on TRM and OS focusing for the first time on a large cohort of alloHCT recipients. In our population of patients with Kp isolates before and after, the burden of GVHD was high. Acute GVHD was linked with pre-transplant Kp infections, suggesting that disruption of intestinal microbiota may be an underlying predisposing condition. Secondary prophylaxis did not improve rates of post-transplant infections but allowed the performance of HCT with an acceptable TRM rate. Importantly, the rate of KCP-Kp infections was alarmingly high in patients post-alloHCT.

Allogeneic HCT recipients represent a unique population threatened by multidrug-resistant bacteria. Earlier agents used for treatment of these infections, such as polymyxins and aminoglycosides, have significant limitations. Therefore, KPC-Kp have emerged as a major cause of bacteremia in patients after autologous or allogeneic HCT. The Italian centers have focused on this problem. In a study of 52 Italian centers, KPC-Kp infections were found in 2% of alloHCT recipients (4). In our population with infections before and after alloHCT, KPC-Kp infection reached 12% of alloHCT recipients.

The importance of these infections in alloHCT is highlighted by their impact on survival rates. Although the majority of reports focus on patients with hematologic malignancies in general, it should be noted that mortality in patients with bacteremia from KPC Enterobacteriaceae exceeds 50% (10). These mortality rates in patients with hematologic malignancies have been attributed to empirical use of prophylaxis and treatment in neutropenic patients (21). These results highlight the importance of antimicrobial stewardship in neutropenic patients (22). Novel combinations of antibiotic agents have been recently approved to address this problem. Ceftazidime-avibactam and ceftolozane-tazobactam are second-generation β-lactam/β-lactamase inhibitor combinations with activity against KPC-Kp (23).

Except for local antibiotic use policies in prophylaxis and treatment, resistance is also influenced by local patterns and infection control measures (24). Recent data from nationwide studies in Greece suggest an increase in colistin resistance rate that is expected to be ameliorated by novel combinations (25–27). Regarding infection control measures, a previous single-center Italian study has focused on HCT recipients (12). This study has shown a reduction of infection-related mortality to 10% in alloHCT recipients, following the introduction of preventive measures. These measures have been actively used in the present study population leading to a similarly low TRM rate. We have not incorporated the term *infection-related mortality*, because of the multiple confounding factors such as GVHD and immunosuppression observed in these patients. Despite the relatively low TRM rate in our population, pre-transplant and post-transplant infections were strongly associated. These data highlight the need for novel antibiotic agents or innovative anti-infectious approaches (28).

Indeed, GVHD rates have been largely ignored by previous studies in the field. GVHD is associated the GI mucosal damage. It is also known that patients with Kp BSIs have concomitant GI colonization (29). In addition, GVHD treatment triggers a higher risk of infections. Furthermore, recent genomic evidence points toward a predictive role of intestinal microbiota as a biomarker of GVHD (30). Our systematic review and meta-analysis confirm for the first time that broad-spectrum antibiotics increase the incidence of acute GVHD based on studies of genomic microbiota diversity (31). Similarly, gut decontamination increases the risk of intestinal GVHD. Therefore, the high rate of acute GVHD and the association with Kp infections are an important finding of the present study.

Our study has some limitations. In particular, our analysis was performed retrospectively in a single-center population transplanted during a 10-year period. It should be noted, however, that patients were transplanted with standard operating procedures of our JACIE-accredited center; data are collected prospectively in our database, and our population consisted only of alloHCT recipients. Furthermore, we decided to approach our research questions from two different clinical angles: one concerning patients with pre-transplant and one concerning those with post-transplant infections. Although there was an overlap of patients between these two groups, we find this separation useful for the clinician, as decisions may be different when facing a patient before or after alloHCT. Another limitation of our study is the lack of data regarding novel antibiotic agents that are expected to improve outcomes in the field.

In conclusion, increased awareness is needed among hematologists and transplant physicians to improve outcomes of patients with Kp isolates. In the era of long-term survival post-alloHCT (32), the burden of infections should be minimized. Larger prospective studies are needed to evaluate novel antibiotic agents or innovative anti-infectious approaches (28) to ameliorate TRM in these patients.

## Data Availability Statement

The datasets presented in this article are not readily available due to patient confidentiality and participant privacy. Requests to access the datasets should be directed to elenicelli@yahoo.gr.

## Ethics Statement

The studies involving human participants were reviewed and approved by G Papanikolaou Hospital Ethics Committee. The patients/participants provided their written informed consent to participate in this study.

## Author Contributions

EG and IS conceived the study idea and design, analyzed the data, wrote and edited the manuscript. TC, SP, AP, EB, and CD collected clinical data and drafted the tables. EK performed laboratory assays. DM, IB, ZB, and VC were involved in patient management and data collection. DS and AA edited and approved the final manuscript. All authors contributed to the article and approved the submitted version.

## Conflict of Interest

The authors declare that the research was conducted in the absence of any commercial or financial relationships that could be construed as a potential conflict of interest.
